# Applicability of *Saccharomyces cerevisiae* Strains for the Production of Fruit Wines Using Cocoa Honey Complemented with Cocoa Pulp

**DOI:** 10.17113/ftb.60.02.22.7285

**Published:** 2022-06

**Authors:** Bárbara Teodora Andrade Koelher, Soraya Maria Moreira de Souza, Andréa Miura da Costa, Elizama Aguiar-Oliveira

**Affiliations:** 1 State University of Santa Cruz (UESC), Rodovia Jorge Amado, km 16, Salobrinho, 45.662-900 Ilhéus, Bahia, Brazil; 2 Executive Commission for Cocoa Cultivation Planning (CEPLAC), Rodovia Jorge Amado, km 22, Primavera, 45.600-970 Ilhéus, Bahia, Brazil

**Keywords:** cocoa honey fermentation, cocoa pulp fermentation, Plackett-Burman design, *S. cerevisiae* L63, sweet wine production

## Abstract

**Research background:**

Cocoa honey and cocoa pulp are both highly appreciated fruit pulp, but until now, cocoa honey has been less processed than cocoa pulp. In this work, we investigate the applicability of *Saccharomyces cerevisiae* strains to ferment cocoa honey complemented with cocoa pulp to obtain fruit wines and improve cocoa honey commercialization.

**Experimental approach:**

The strain, previously isolated from *cachaçaria* distilleries in Brazil, was selected based on its fermentation performance. The following conditions for fermentation with *S. cerevisiae* L63 were then studied: volume fraction of cocoa honey (*φ*_CH_) complemented with cocoa pulp, sucrose addition (*γ*_suc_), temperature (*t*) and inoculum size (*N*_o_). The best conditions were applied in order to obtain fermentation profiles.

**Results and conclusions:**

*S. cerevisiae* L63 (*N*_o_=10^7^–10^8^ cell/mL) is capable of fermenting *φ*_CH_=90 and 80% for 24 or 48 h with *γ*_suc_=50 and 100 g/L at *t*=28–30 °C resulting in wines with ethanol volume fractions from 8 to 14%. Additionally, the wine produced from *φ*_CH_=90% had lower residual sugar concentration (<35 g/L) than the wine produced from *φ*_CH_=80% (~79 g/L) which could be classified as a sweet wine. In general, *S. cerevisiae* L63 resulted in a similar fermentation performance as a commercial strain tested, indicating its potential for fruit pulp fermentation.

**Novelty and scientific contribution:**

*Saccharomyces cerevisiae* L63 can ferment cocoa honey complemented with cocoa pulp to produce fruit wines with good commercial potential, which may also benefit small cocoa producers by presenting a product with greater added value.

## INTRODUCTION

The information obtained through recent investigation about fermentation of different fruit pulp (*1*-*3*) is valuable since it allows a better understanding of the processes crucial for obtaining products of higher quality and competitiveness. Furthermore, it can contribute to the reduction of postharvest losses, which are common in tropical countries. A few examples of tropical fruits used to obtain fruit wines are: yellow mombin (*4*), pineapple (*5*), cashew (*6*), mango (*7*), guava (*8*) and carambola (*9*). Fermentation can be performed by different microorganisms; however, *Saccharomyces cerevisiae* has been the most traditionally applied yeast in fruit wine production due to its versatility (*10*-*12*).

Cocoa (*Theobroma cacao* L.) is known worldwide for its beans that are used in the production of chocolate and cocoa butter (*13*). Cocoa pulp is one of the by-products resulting from the processing of its fruit and is frequently used in the preparation of juice, but it has also been used for the production of fruit wine (*14*-*16*). Another by-product of cocoa processing is a mucilaginous pulp called cocoa honey. This pulp is obtained (in much smaller volume) after the initial pulping and it drips from the cocoa beans just before they start to ferment. Both cocoa pulp and honey are rich in nutrients such as sugars, fiber and bioactive compounds (*17*, *18*). The pectin content in cocoa honey is higher than in the pulp, reaching ~2.5% (*m/V*) (*19*). Cocoa honey has more often been used for the production of artisanal jelly (*19*, *20*) and for fresh consumption, basically due to its higher pectin content and its higher perishability, in comparison to cocoa pulp. However, it is known that cocoa honey can be used for the production of fruit wine, as observed previously by Leite *et al.* (*17*) and Magalhães-Guedes *et al.* (*21*). Consequently, the combined application of cocoa honey and pulp can result in distinguished fruit wines.

Thus, in view of the importance of cocoa cultivation for the southern region of Bahia (Brazil) and in order to stimulate the use of cocoa honey to obtain products of added value, this work aims to select a strain of *S. cerevisiae* (previously isolated from different cachaça distilleries) and define the best conditions for the selected strain, *S. cerevisiae* L63, to ferment cocoa honey complemented with cocoa pulp for obtaining fruit wines.

## MATERIALS AND METHODS

### Materials

The cocoa honey was donated by the company Du Kakau (Una, Bahia, Brazil). According to the manufacturer, it was obtained by cold pressing and filtration, without added sugars and/or preservatives. The cocoa pulp was produced by the company Sabiá (Ilhéus, Bahia, Brazil). Both substrates were kept frozen until use. The citrus pectin was donated by the company Herbstreith & Fox (Neuenbürg, Germany). All other chemicals were purchased locally from reliable suppliers.

#### Microorganisms: maintenance, cultivation and inoculum preparation

The strains of *Saccharomyces cerevisiae* investigated in this research (L13, L37, L63 and L67) were previously isolated and identified by Silva *et al.* (*18*) and characterized according to pectinase activity by Carvalho *et al*. (*22*). They belong to the culture stock of the Applied Microbiology Laboratory (LABMA/UESC, Ilhéus, Bahia, Brazil) and they are preserved in a 15% (*m*/*V*) glycerol solution at -80 °C. A lyophilized commercial yeast was also used for performance comparison: *S. cerevisiae* CA-11 (recommended for the production of cachaça, a Brazilian distilled spirit made from sugarcane), which was donated by the company LNF (Bento Gonçalves, Rio Grande do Sul, Brazil).

The stock cellular solutions were obtained by cultivating each strain in 25 mL of Sabouraud broth at 28 °C for 48 h (SL-200; Solab, Piracicaba, São Paulo, Brazil). After cultivation, 100-µL aliquots were spread over the surface of the same solid medium that was incubated once again at 28 °C for 24 h. Then, the cells grown on the surface of the Petri dishes were scraped and rinsed with 2-mL aliquots of sterile distilled water. Total cell count in the collected volume was determined in a Newbauer chamber. This value was used to obtain the initial cell number *N*_o_ (cell/mL), labeled inoculum 1, which was then used in the preliminary tests and the study of fermentation conditions. For the activation and inoculum preparation of the commercial strain, 3 g of the lyophilized cells were used following the same procedure.

Inoculum 2 was adapted to the fermentation must by using approx. 30 mL of inoculum 1, 50 mL of cocoa honey and 2.5 g of sucrose, with sterile distilled water added until reaching a volume of 100 mL. Cultivation occurred at 30 °C for 12 h (SP-222; SP Labor, Presidente Prudente, São Paulo, Brazil), without stirring, and then the cell mass was separated by centrifugation (15 000×*g* for 5 min; 16R; Thermo Scientific, Jundiaí, São Paulo, Brazil) and the supernatant was discarded. The cells were resuspended in 15 mL of sterile distilled water and the total cell count was determined as described above. The same procedure was followed for strain CA-11.

#### Selection of the strain

The strains (L13, L37, L63 and L67) were cultivated in 250-mL Erlenmeyer flasks containing 150 mL of culture medium. The performance of each strain during incubation was observed for 4–5 days, including cell count (*N*/(cell/mL)), total soluble solid content (g/100 g), pH and flocculation (visual analysis), while ethanol volume fraction (%) was analyzed only at the end of fermentation. Initially, the strains were cultivated in solutions of commercial pectin (20 g/L) and sucrose (100 g/L) in a phosphate buffer (0.5 M at pH=4.0) with *N*_o_ around 1·10^7^–2·10^7^ cell/mL. Incubation was carried out at 30 °C and agitation speed of 150 rpm (SP-222; SP Labor). Then, the cultivation of strains L13 and L63 was evaluated in pure cocoa honey (*φ*_CH_=100%) at initial conditions of pH=3.43 and 16.8 °Brix, under the same temperature and *N*_o_, but without sucrose and agitation.

#### Experimental design for fermentation conditions

The best conditions for fermentation of cocoa honey and cocoa pulp by the yeast *S. cerevisiae* L63 were investigated applying the statistical tool of experimental design (*23*) with a Plackett-Burman matrix (PB8). For a total volume of 200 mL of medium, the independent variables (factors) and their lowest (-1) and highest (+1) levels were: volume fractions of cocoa honey (with or without cocoa pulp) (*φ*_CH_=80−100%), sucrose concentration (*γ*_suc_=0−100 g/L), temperature (*t*=28−32 °C) and initial cell count (*N*_o_=10^6^−10^8^ cell/mL). The dependent variables (responses) investigated over 72 h of fermentation were: ethanol volume fraction (%), cell count (cell/mL), pH, total soluble solid content (g/100 g) and absorbance (*A*) read at 600 nm. In order to use the same cocoa honey batch for all of the PB8 experiments, it was not possible to perform the central points in triplicate but only in one replicate.

#### Statistical analysis

The responses obtained with the experimental design were used in the effects analysis (*23*) performed at 85% confidence using the statistical software Protimiza Experimental Design (*24*). In this analysis, the individual effects of each factor were considered statistically significant at p<0.15, which indicates that by increasing the factor from its lowest to its highest level, it is possible to obtain a statistically significant alteration (positive or negative) in the average response.

#### Fermentation profiles

The concentrations of sugar, organic acid and alcohol (*γ*), number of cells (*N*), total soluble solids, pH and *A* were determined throughout the fermentation (*V*=200 mL) at 28 °C and *N*_o_=6.5·10^7^ cell/mL, with samples being collected between 3 and 48 h. For both values of *φ*_CH_=90 and 80% fermentations were carried out with *γ*_suc_=50 and 100 g/L for 24 h and 48 h, respectively. For comparison, the commercial yeast strain was used under the same conditions. Cocoa honey, cocoa pulp and the volume fractions of both (without the addition of sucrose) were analyzed by chromatography for sugar, organic acid and alcohol concentrations. Due to batch limitation of cocoa honey and cocoa pulp, it was only possible to perform one replicate in the analysis.

#### Physicochemical analysis

The total soluble solids were determined with a digital refractometer (model RHB90; Akso Produtos Eletrônicos Ltda, São Leopoldo, Rio Grande do Sul, Brazil) on a °Brix scale and expressed in g/100 g, and pH was determined with a pH meter (model PHS3BW; Bel Engineering®, Rio de Janeiro, Rio de Janeiro, Brazil). The ethanol volume fraction (%) was determined with a refractometer (RETK-75; Tekcoplus Ltd., Hong Kong, PR China). To assess the clarification of the fermented must, the absorbance (*A*) was read at 600 nm in a spectrophotometer (SP 2000 UV; Bel Engineering®).

Mass concentrations (g/L) of sucrose, glucose, fructose, ethanol, methanol, glycerol and citric, lactic and acetic acids were determined by high-performance liquid chromatography (HPLC, Hitachi Primaide; Tokyo, Japan) with an ion exchange column (Aminex HPX-87H, 300 mm×7.8 mm; Bio-Rad, Hercules, CA, USA) and an infrared detector (K-9800; Knauer, Berlin, Germany). The samples were diluted 20 times in ultrapure water and filtered in a polyvinylidene difluoride (PVDF) membrane filter (0.45 μm) with an injection volume of 20 µL. For the analytical run, an isocratic system was used with the mobile phase of H_2_SO_4_ (0.01 M) and a flow rate of 0.6 mL/min at room temperature (approx. 26 °C) and a 20-minute running time. The acquisition and integration of the peaks was performed using the Star Chromatography Workstation v. 6.0 software (*25*) based on standard curves previously prepared with chromatographic standards (Sigma-Aldrich, Merck, St. Louis, MO, USA).

## RESULTS AND DISCUSSION

### Strain selection

The fermentation performance of the four strains of *Saccharomyces cerevisiae* (L13, L37, L63 and L67) in a medium containing pectin and sucrose (to mimic the concentration in cocoa honey *in natura*) was evaluated over 98 h and, in general, the pH remained stable, varying between 3.88 and 3.97 at the beginning and between 3.68 and 3.82 at the end of fermentation. These results are in line with what has been reported by other researchers, for example, with fermentation of pear and kiwi pulp by *S. cerevisiae* WLS2 (*26*). Strains L13 and L63 showed the greatest and strain L67 the lowest reduction of total soluble solids throughout cultivation. The cell count (*N*) ​​remained in the range of 10^7^ cell/mL, and a stronger flocculation with strains L37 and L67 was observed, indicating that flocculation was induced by agitation of these two strains. Agitation is one of the factors that favours cell-to-cell adhesion and promotes flocculation (*27*). Although flocculation is appreciated in the industrial production of cachaça (since it facilitates the separation of cells at the end of the process, without the need for centrifugation or filtration), in these preliminary tests, flocculation was observed to be detrimental to the performance of fermentations (especially for *φ*_ethanol_). Strains L13 and L63 showed more stable values of *N* and total soluble solids (probably due to their reduced flocculation) and, for this reason, these two strains were selected to continue the study. Then, static fermentations were carried out for 93 h with cocoa honey, and the ethanol volume fractions (*φ*_ethanol_) obtained at the end of fermentation were similar: with L13 it was 8.78% and with L63 it was 8.67%. Under these conditions, it was observed (for both strains) that the maximum *N* was around 10^8^ cell/mL (between 69 and 72 h). In addition, pH showed little variation around 3.5 and the greatest reduction (approx. 65%) in total soluble solid content was observed around 42 h.

**Fig. S1:**
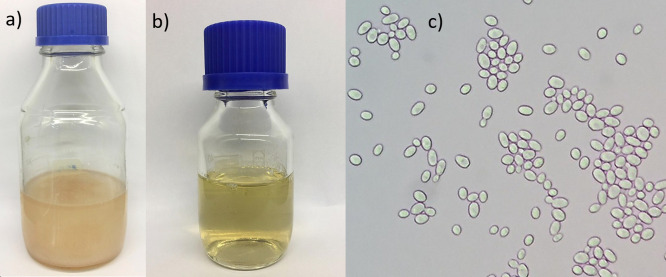
Cocoa honey: a) before (*A*_600 nm_=1.165) and b) after (*A*_600 nm_=0.084) fermentation by c) *S. cerevisiae* L63 (observed under a microscope at 400× magnification)

Based on a previous study by Carvalho *et al.* (*22*), where the highest production of pectinases by L63 strain was reported (up to 3.5 times more pectinases than strain L13, for example), this strain was chosen for cocoa honey fermentation for the purpose of clarification due to the hydrolysis of pectin. For the simultaneous fermentation and clarification of cocoa pulp, for example, commercial pectinases and cellulases were added together with strain CA-11 in the work of Duarte *et al.* (*16*). Dias *et al.* (*14*) also used pectinases as a pretreatment of cocoa pulp for fermentation. To exemplify the clarification of the medium, Fig. S1 shows a comparison of cocoa honey before (Fig. S1a) and after (Fig. S1b) fermentation by strain L63 (Fig. S1c). The obtained results are promising; however, it is necessary to emphasize that strains L13, L37 and L67 also have good potential for fermentation of fruit pulp and should be better investigated in subsequent studies.

#### Study of the best conditions for fermentation

Fermentations were carried out and the obtained *φ*_ethanol_ and *N* were considered the main responses to be analyzed, while pH, total soluble solids (TSS) and *A* were analyzed as complementary responses at the beginning (0 h) and at the end of fermentation (72 h). From the results presented in Table 1, it is possible to observe that the highest obtained *φ*_ethanol_ corresponded to run 2 (72 h) and run 6 (48 and 72 h). However, it is also important to observe the *φ*_ethanol_ value of run 9, which was obtained in a shorter time (24 h) and, consequently, represents a higher productivity. These alcohol volume fractions are in accordance with the range stipulated for fruit wines in Brazil: 4–14% (*28*). For the response *N*, it is possible to observe that regardless of *N*_o_, its value did not exceed the factor of 10^8^ (Table 1), which was also confirmed by Dias *et al.* (*14*). Thus, a range of *N*_o_=10^7^–10^8^ cell/mL is suggested considering possible difficulties in larger scales for the preparation of inoculum solution and cell count. A similar inoculum (10^8^ cell/mL) was used for fermentation of cocoa pulp by Duarte *et al.* (*16*). In relation to the pH values, small variations were once again observed during fermentation (Table 1). The clarification of the must can also be confirmed due to the reduction of the total soluble solid content and *A* throughout fermentation; the highest reductions (>70%) of total soluble solid content were obtained in runs 5 and 9, and for more than half of the performed runs, *A* was reduced in more than 95% (Table 1). The clarification associated with fermentation can be understood, for example, when related to the consumption of sugar, due to cell growth, and/or enzymatic hydrolysis of pectin.

**Table 1:** Coded Plackett-Burman matrix (PB8) for the study of fermentation and clarification of cocoa honey and cocoa pulp by *Saccharomyces cerevisiae* L63 with the factors: volume fraction of cocoa honey (*φ*_CH_), sucrose concentration (*γ*_suc_), temperature (*t*) and inoculum (*N*_o_) and the responses: ethanol volume fraction (*φ*_ethanol_), cell count (*N*), pH, total soluble solid content (TSS) and absorbance (*A*_600 nm_). Real values of factors are presented in parentheses

Run	Factor				Response											
*φ*_ethanol_/%			*N*/(cell/mL)			pH		*w*(TSS)/(g/100 g)		*A*_600 nm_	
*φ*_CH_/%	*γ*_suc_/(g/L)	*t*/°C	*N*_o_/(cell/mL)	Time/h											
				24	48	72	24	48	72	0	72	0	72	0	72
1	+1 (100)	-1 (0)	-1 (28)	+1 (10^8^)	8.30	9.00	9.10	3.0·10^8^	2.3·10^8^	2.5·10^8^	3.73	3.40	14.4	4.8	1.165	0.084
2	+1 (100)	+1 (100)	-1 (28)	-1 (10^6^)	3.70	9.60	13.60	1.0·10^8^	1.6·10^8^	1.2·10^8^	3.73	3.38	23.3	8.6	0.957	0.081
3	+1 (100)	+1 (100)	+1 (36)	-1 (10^6^)	5.00	10.20	12.60	5.7·10^7^	8.6·10^7^	1.1·10^8^	3.71	3.44	22.9	9.2	1.040	0.113
4	-1 (80)	+1 (100)	+1 (36)	+1 (10^8^)	4.90	9.80	12.30	3.3·10^7^	6.8·10^7^	5.7·10^7^	3.53	3.57	23.2	8.9	1.786	0.082
5	+1 (100)	-1 (0)	+1 (36)	+1 (10^8^)	8.90	9.00	9.10	1.9·10^8^	1.8·10^8^	1.7·10^8^	3.63	3.50	15.6	4.0	1.165	0.092
6	-1 (80)	+1 (100)	-1 (28)	+1 (10^8^)	9.50	13.50	14.00	1.1·10^8^	1.8·10^8^	1.6·10^8^	3.73	3.60	19.8	6.2	1.786	0.073
7	-1 (80)	-1 (0)	+1 (36)	-1 (10^6^)	1.80	6.70	8.50	1.2·10^7^	3.6·10^7^	1.6·10^7^	3.59	3.72	15.0	5.0	2.154	0.080
8	-1 (80)	-1 (0)	-1 (28)	-1 (10^6^)	0.00	0.50	6.50	1.8·10^6^	1.3·10^7^	7.0·10^7^	3.54	3.66	15.1	7.2	2.150	0.066
9	0 (90)	0 (50)	0 (32)	0 (10^7^)	10.50	11.10	11.20	1.9·10^8^	1.9·10^8^	2.2·10^8^	3.60	3.71	19.2	5.2	1.370	0.068

*V*_total_=200 mL

Considering the analysis of effects (Table 2) for the response *N*, the only factor with a positive and statistically significant effect (p<0.15) at all times was *N*_o_, and only at 24 h, it was *φ*_CH_. For *φ*_ethanol_, *N*_o_ was also statistically significant and positive for the first part of fermentation (24 and 48 h), with its highest effect in the first 24 h. The concentration of sucrose (*γ*_suc_) also had a positive and statistically significant effect in the second half of fermentation (48 and 72 h). In general, this analysis suggests that *N* can be increased in the first 24-48 h of fermentation with the increase of *γ*_suc_, *N*_o_ and *φ*_CH_, as demonstrated in the obtained responses in Table 1. Additionally, in order to increase *φ*_ethanol_, fermentation can be extended up to 48 h with a higher *γ*_suc_ value. Obtaining a fermented product with higher ethanol volume fraction is interesting as it can be used, for example, to obtain a distilled spirit, denominated in Brazil as aguardente de fruta (*28*).

**Table 2:** Effect analysis of the factors: volume fraction of cocoa honey (*φ*_CH_), sucrose concentration (*γ*_suc_), temperature (*t*) and innoculum (*N*_o_) for the responses: alcohol volume fraction (*φ*_ethanol_) and cell count (*N*)

Response: *φ*_ethanol_/%	Time/h											
24				48				72			
Term	Effect	S.D.	*t*(4)	p-value	Effect	S.D.	*t*(4)	p-value	Effect	S.D.	*t*(4)	p-value
Mean	4.79	0.90	5.35	0.0059*	8.82	0.93	9.52	0.0007*	10.77	0.34	31.92	<0.0001*
*φ*_CH_/%	2.43	1.90	1.28	0.2710	1.83	1.96	0.93	0.4055	0.78	0.72	1.08	0.3396
*γ*_suc_/(g/L)	1.03	1.90	0.54	0.6183	4.48	1.96	2.28	0.0850*	4.83	0.72	6.74	0.0025*
*t*/°C	-0.23	1.90	-0.12	0.9115	0.78	1.96	0.39	0.7134	-0.18	0.72	-0.24	0.8188
*N*_o_/(cell/mL)	5.28	1.90	2.78	0.0500*	3.58	1.96	1.82	0.1429*	0.83	0.72	1.15	0.3131
Response: *N*/(cell/mL)	Time/h											
	24				48				72			
Term	Effect	S.D.	*t*(4)	p-value	Effect	S.D.	*t*(4)	p-value	Effect	S.D.	*t*(4)	p-value
Mean	9.9·10^7^	2.2·10^7^	4.44	0.0113*	1.0·10^8^	2.2·10^7^	4.85	0.0083*	1.1·10^8^	2.7·10^7^	4.38	0.0119*
*φ*_CH_/%	9.7·10^7^	4.7·10^7^	2.06	0.1085*	5.0·10^7^	4.7·10^7^	1.05	0.3534	5.7·10^7^	5.6·10^7^	1.01	0.3714
*γ*_suc_/(g/L)	-7.5·10^7^	4.7·10^7^	-1.59	0.1861	-3.0·10^7^	4,7·10^7^	-0.64	0.5539	-4.4·10^7^	5.6·10^7^	-0.78	0.4770
*t*/°C	-3.0·10^7^	4.7·10^7^	-0.64	0.5594	-1.3·10^7^	4.7·10^7^	-0.28	0.7894	-3.2·10^7^	5.6·10^7^	-0.56	0.6022
*N*_o_/(cell/mL)	1.4·10^8^	4.7·10^7^	2.95	0.0418*	1.3·10^8^	4.7·10^7^	2.73	0.0526*	1.0·10^8^	5.6·10^7^	1.94	0.1246*

*Statistically significant effect with 85% of confidence (p<0.15). *t*(4)= calculated *t*-test (degree of freedom). S.D.=standard deviation. *V*_total_=200 mL

Considering the best *φ*_ethanol_ values (Table 1), run 9 also gave (within 24 h) pH, TSS and *A* values of: 3.46, 5.4 g/100 g and 0.078, respectively, and run 6 (within 48 h): 3.62, 7.0 g/100 g and 0.088, respectively. Between these two fermentations, run 9 was carried out with a higher *φ*_CH_ and lower *γ*_suc_ than run 6. The results also indicate that increasing the fermentation time up to 72 h was not advantageous for *φ*_ethanol_ since the observed increments did not justify extending the fermentation time to more than 48 h. Runs 6 and 9 were both performed with initial TSS content around 19 g/100 g, which is close to the value used by Leite *et al.* (*17*) with cocoa honey. For fermentation of other types of fruit pulp with *S. cerevisiae,* values from 16 to 20 g/100 g have been reported (*1*, *29*).

In general, the obtained results are in accordance with the results of cocoa honey and cocoa pulp fermentations carried out by other researchers. The fermentation of cocoa honey with a commercial strain (*S. cerevisiae* AWRI726) was also evaluated by Magalhães-Guedes *et al.* (*21*), considering three factors: temperature, total soluble solid content and fermentation time. In this example, the authors obtained the highest alcohol volume fraction (15%) at 25 °C, 13 g/100 g (with sucrose addition) and a longer time (96 h). Furthermore, an increase in temperature to 28 °C and time to 112 h was favorable for fermentation. In a complementary study, this same research group obtained an alcohol content of 16% when fermenting cocoa honey with the same strain but at 20 °C for 144 h (*17*).

In another example, fermentation of cocoa pulp by *S. cerevisiae* UFLA CA 1162 resulted in an alcohol content of about 8% and fermentation was carried out for 30 days at 22 °C followed by a period of wine maturation (*16*). Also, cocoa pulp fermentation by *S. cerevisiae* CA1183 resulted in approx. 11% ethanol and pH=3.7, after 64 h at 25 °C with an additional period of maturation (*14*). An alcohol volume fraction of approx. 10% and pH=3.6 were obtained when using 80% cocoa pulp (previously clarified with pectinases) complemented with *Hibiscus sabdariffa* L. extract fermented at 30 °C for 168 h by *S. cerevisiae* var. *bayanus* (*15*).

As temperature (*t*) was not statistically significant (p>0.15) in the analyzed range for the two responses (Table 2), the lowest value (28 °C) was chosen. However, based on the preliminary tests and other published results with *S. cerevisiae* (*6*, *7*, *15*, *29*), the range of 28–30 °C can be suggested as well. As the increase in *φ*_CH_ up to 100% slowed down *N*, but had no effect on *φ*_ethanol_, both *φ*_CH_ (90 and 80%) were chosen due to the possibility of achieving a different sensorial profile of fruit wines. Additionally, 24 h and *γ*_suc_=50 g/L were selected as suitable for obtaining a fruit wine, while 48 h and *γ*_suc_=100 g/L were selected for obtaining higher alcohol volume fractions (*e.g.* for distillation).

#### Fermentation profiles and composition of fruit wines

**Fig. 1:**
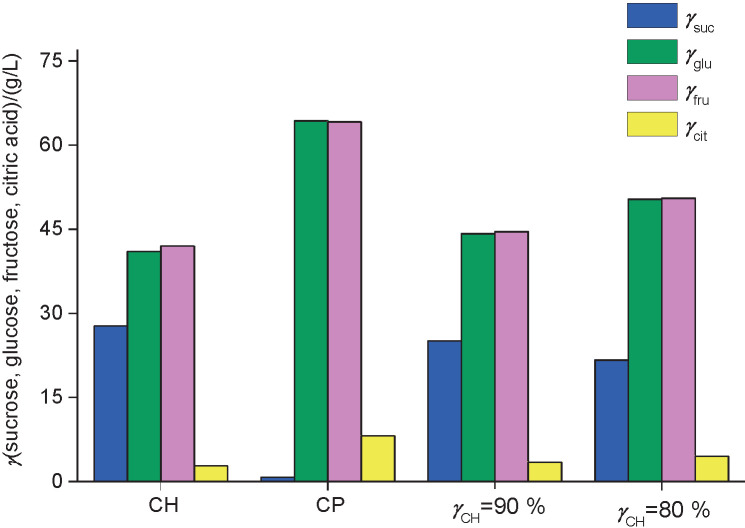
Mass concentrations (g/L) of sucrose (*γ*_suc_), glucose (*γ*_glu_), fructose (*γ*_fru_) and citric acid (*γ*_cit_) in cocoa honey (CH), cocoa pulp (CP) and the volume fractions (*φ*_CH_) of 90 and 80% of cocoa honey added to cocoa pulp

Fig. 1 shows the concentrations of sugar and citric acid in cocoa honey and cocoa pulp, and according to the results, cocoa honey had a higher concentration of sucrose (27.75 g/L), and cocoa pulp had higher concentrations of citric acid (*γ*_cit_=8.17 g/L), fructose (*γ*_fru_=64.12 g/L) and glucose (*γ*_glu_=64.28 g/L). Thus, the two selected *φ*_CH_ for the fermentation of must can be easily understood as a reflection of their individual compositions (Fig. 1).

**Fig. 2:**
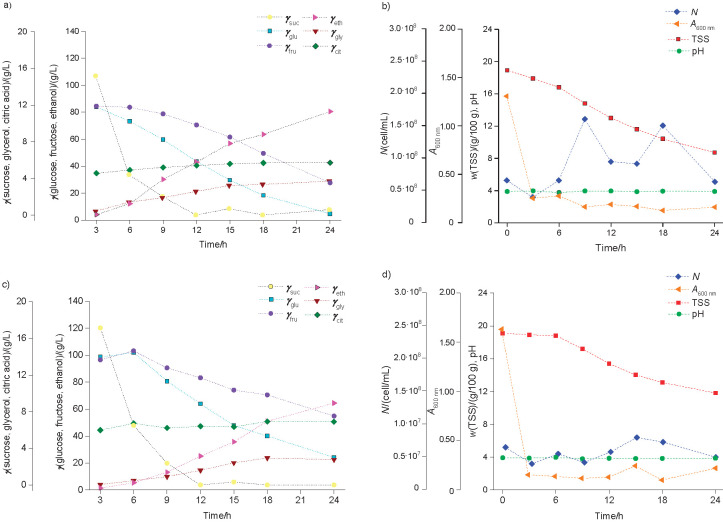
Fermentation profiles of *Saccharomyces cerevisiae* L63 in cocoa honey at: a) and b) 90%, and c) and d) 80% added cocoa pulp and 50 g/L of sucrose for 24 h. Concentrations (g/L) of sucrose (*γ*_suc_), glucose (*γ*_glu_), fructose (*γ*_fru_), glycerol (*γ*_gly_), citric acid (*γ*_cit_) and ethanol (*γ*_eth_) are presented in a) and c). Cell count (*N*), absorbance (*A*), total soluble solids (TSS) and pH are presented in b) and d)

**Fig. 3:**
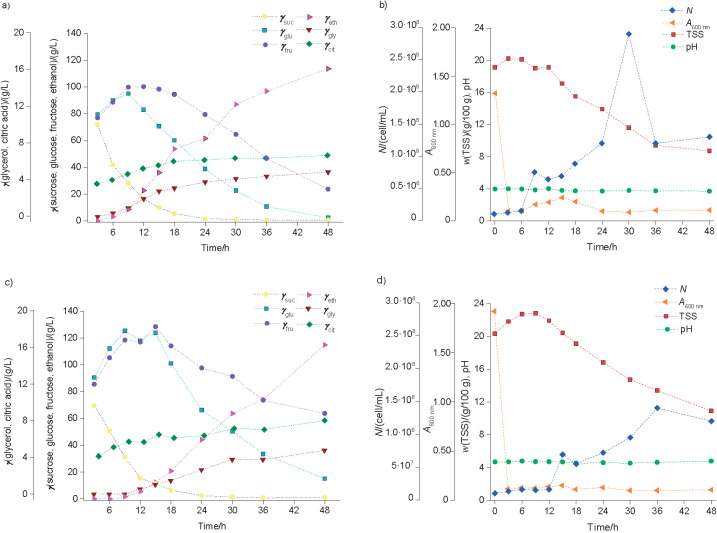
Fermentation profiles of *S. cerevisiae* L63 in cocoa honey at: a) and b) 90% and c) and d) 80% added cocoa pulp and 100 g/L of sucrose for 48 h. Concentrations (g/L) of sucrose (*γ*_suc_), glucose (*γ*_glu_), fructose (*γ*_fru_), glycerol (*γ*_gly_), citric acid (*γ*_cit_) and ethanol (*γ*_eth_) are presented in a) and c). Cell count (*N*), absorbance (*A*), total soluble solids (TSS) and pH are presented in b) and d)

Fermentations conducted for 24 h (Fig. 2) and 48 h (Fig. 3) with both *φ*_CH_ were investigated to determine the concentrations of sugars, alcohols and acids (Figs. 2a, 2c, 3a and 3c) and *N*, TSS content, pH and *A* (Figs. 2b, 2d, 3b and 3d). For these fermentations, it was not possible to obtain *N*_o_=10^8^ cell/mL as desired; however, the actual value employed (6.5·10^7^ cell/mL) was within the suggested range for inoculum cell count. In general, the obtained fermentation profiles indicated an increase in *φ*_ethanol_ and *N* associated with the consumption of sugars and simultaneous clarification, which is in agreement with other studies (*16*, *17*). Table 3 also shows the compilation of the final values for fruit wines fermented with strain L63 in comparison with the commercial strain for both *φ*_CH_ (90 and 80%) and times (24 and 48 h).

**Table 3:** Final parameters of fruit wines obtained from volume fractions of cocoa honey 90 and 80% complemented with cocoa pulp fermented by *S. cerevisiae* strains L63 and CA-11 at 28 °C for 24 h (with 50 g/L sucrose) and 48 h (with 100 g/L sucrose)

Parameter	*S. cerevisiae* L63				*S. cerevisiae* CA-11			
*φ*_CH_/%							
90	80	90	80	90	80	90	80
Time/h							
24		48		24		48	
*φ*_ethanol_/%	10.20	8.20	14.40	14.60	10.70	8.10	15.90	14.50
pH	3.89	3.85	3.69	3.98	3.85	3.90	3.74	3.76
*w*(TSS)/(g/100 g)	8.7	11.8	8.7	10.9	13.0	12.8	8.4	7.1
*A*_600 nm_	0.161	0.218	0.110	0.105	0.139	0.076	0.096	0.066
*γ*/(g/L)								
Sucrose	0.58	n.d.	0.74	1.01	1.24	1.56	0.88	n.d.
Fructose	27.67	54.90	23.68	63.74	65.23	62.34	9.31	8.48
Glucose	4.60	24.16	2.39	15.08	22.36	34.23	0.90	0.78
Acetic acid	n.d.	n.d.	n.d.	0.97	n.d.	n.d.	n.d.	n.d.
Citric acid	5.66	6.86	6.63	8.04	6.0	5.14	6.52	6.0
Lactic acid	0.21	n.d.	n.d.	n.d.	n.d.	n.d.	1.00	n.d.
Methanol	n.d.	n.d.	n.d.	n.d.	n.d.	n.d.	n.d.	n.d.
Glycerol	3.70	2.72	4.88	4.77	5.14	3.25	6.36	4.81

*V*_total_=200 mL. TSS=total soluble solids, n.d.=not detected

In regard to the fermentations performed for 24 h with *γ_suc_*=50 g/L, it was observed (Figs. 2a and 2c) that for both *φ*_CH_ there was a marked decrease in *γ*_suc_ in the first 12 h of fermentation and lower reductions of glucose (*γ*_glu_) and fructose (*γ*_fru_) after 6 h of fermentation. It is also possible to infer the preferential consumption of glucose by *S. cerevisiae* L63, since *γ*_fru_ remained higher than *γ*_glu_ until the end of fermentation. In the cocoa honey fermentation evaluated by Leite *et al.* (*17*), for example, total sugar concentration was reduced from 186.78 to 5.02 g/L after 240 h of fermentation with a residual fructose concentration of 4.21 g/L. In this work, the must with *φ*_CH_=90% resulted in a total reduction of 54% of total soluble solid content, a better result than with *φ*_CH_=80%, where total reduction was 38%. In addition, the highest reduction (91%) of *A* occurred in the first 3 h of fermentation with *φ*_CH_=80%, but the lowest *A* value was achieved with *φ*_CH_=90% (greatest clarification) at the end of 24 h (Figs. 2b and 2d). Considering the variability, the increase in *N* was once again greater at higher volume fraction of cocoa honey than of cocoa pulp. Also, the pH and citric acid (*γ*_cit_) values varied little over time (Figs. 2b and 2d) and methanol, acetic and lactic acid were not identified in any of the fermentations.

After 24 h of fermentation, the wine obtained from *φ*_CH_=90%(Table 3) had a higher alcohol volume fraction, while the wine obtained from *φ*_CH_=80% resulted in a higher concentration of residual sugar (79.06 g/L), which is in accordance with the classification of sweet wine based on the Brazilian legislation (*28*). Regarding the fruit wine fermented with the commercial strain for 24 h, compositions were similar, especially in relation to *φ*_ethanol_. However, higher concentrations of residual sugar (>88 g/L) were obtained (Table 3).

Similar trend was observed for the fermentations conducted for 48 h with *γ*_suc_=100 g/L, where sucrose was almost entirely consumed around 24 h and the concentrations of *γ*_glu_ and *γ*_fru_ showed a decrease from 12–15 h (Figs. 3a and 3c). The wine obtained from *φ*_CH_=90% also had a reduced *γ*_glu_ at the end of fermentation (Table 3). After 48 h of fermentation, the volume fractions of ethanol in both fruit wines increased with time (Figs. 3a and 3c) and the final values of *φ*_ethanol_ (Table 3) were similar. Once again, the wine obtained from *φ*_CH_=80% had a higher concentration of residual sugar (79.83 g/L). The TSS content (Figs. 3b and 3d) increased in the first 12 h of fermentation (as a consequence of the change of sugar concentrations) followed by a constant decrease, representing 65–67% reduction until the end of fermentation. Clarification was also achieved since the *A* values showed reductions between 94–96% at the end of fermentation (Figs. 3b and 3d). In comparison with the fruit wine fermented for 48 h using the commercial strain CA-11 (Table 3), it is also possible to observe similar values of alcohol but much lower values of residual sugars (<11 g/L).

The citric acid (*γ*_cit_) concentrations increased throughout both fermentations for 24 (Figs. 2a and 2c) and 48 h (Fig. 3a and 3c). During the former, the increments were between 15–25%, and during the latter, larger increments (87–94%) were observed. In general, lactic acid, acetic acid and methanol were not detected, except for the small concentrations of acetic acid in the wine obtained from *φ*_CH_=80% with the strain L63, and lactic acid in the wine obtained from *φ*_CH_=90% with the strain CA-11 and after 48 h of fermentation (Table 3). The organic acids naturally found in fruit wines are fundamental to the unique sensory quality of each beverage (*16*). These acids can come from the used fruit pulp or they can be produced during fermentation. Citric acid is an example that fits these two situations and its presence is generally valued for sensory aspects. However, the presence of lactic and/or acetic acid, for example, can indicate contamination of the fermentation process and is a good indicator of quality (*30*) or it can be detected as a result of fermentation. For example, the cocoa pulp wine obtained by Dias *et al.* (*14*) had 5.5 g/L citric acid, 1.1 g/L acetic acid, but it did not contain lactic acid. Longer fermentations of fruit pulp generally require the addition of sulfur dioxide (up to 100–200 mg/L of free SO_2_) in the form of dipotassium disulfite to inhibit bacterial growth (*14*, *16*, *17*). This was not applied in this study, but it is also recommended for scaled up fermentations.

In fruit wine production, the presence of glycerol also contributes to sensory characteristics such as sweetness and fullness, as mentioned by Dias *et al.* (*14*), who obtained glycerol concentrations of 5.53–9.0 g/L in a cocoa pulp wine. In this study, the glycerol concentrations increased with time (Figs. 2a, 2c, 3a and 3c) and the final concentrations obtained for the wines fermented for 48 h (Table 3) are within the range declared by Gamella *et al.* (*31*) for dry wines (4–10 g/L); the wines fermented for 24 h had lower concentrations of glycerol (Table 3). Interestingly, different *Saccharomyces* strains have been investigated in order to obtain wines with low-ethanol content, higher smoothness and a greater capacity to produce glycerol (*32*).

## CONCLUSIONS

The presented data lead to a conclusion that *Saccharomyces cerevisiae* L63 demonstrated not only good fermentation performance in musts composed of cocoa honey and cocoa pulp, but also a good ability to promote clarification during fermentation, thus resulting in fruit wines with a crystalline aspect and basic chemical composition in accordance with the current Brazilian legislation. The obtained results also confirm the versatility and the potential when using a volume fraction of cocoa honey and cocoa pulp. It is important to perform future studies (different strains, sensorial analysis, *etc*.) to deepen the knowledge about the use of the pulp of different fruits to obtain alcoholic beverages, such as fruit wines as well as distilled spirits. Nonconventional fruit pulp, as a substrate for fermentation and distillation, can definitely contribute to obtaining promising beverages by adding valuable sensorial aspects to the final product compared to sugarcane, apple or grape, for example.
